# Supplementary material for: A Bacterium Derived from the Ovary of the Black Soldier Fly (Hermetia illucens) Attract Oviposition of the Host
Source: Biology (Basel). 2025 Aug 22;14(9):1107. doi: 10.3390/biology14091107 (PMC12467855; doi:10.3390/biology14091107)
Supplement: Supplementary file 1 [file biology-14-01107-s001.zip › Figure S1 Morphology and Identification of Six Bacterial Strains.pdf]

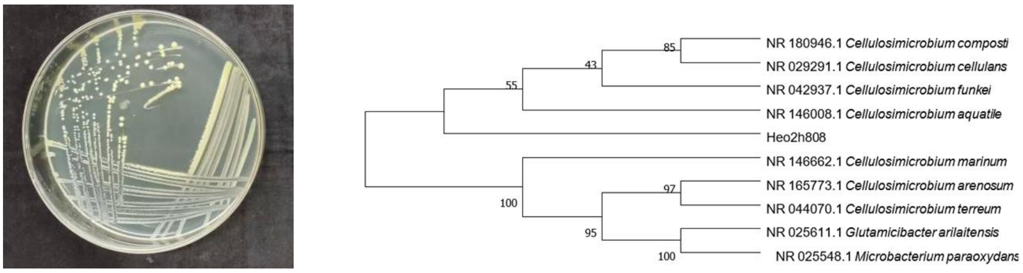

Cellulosimicrobium sp. Heo2h808

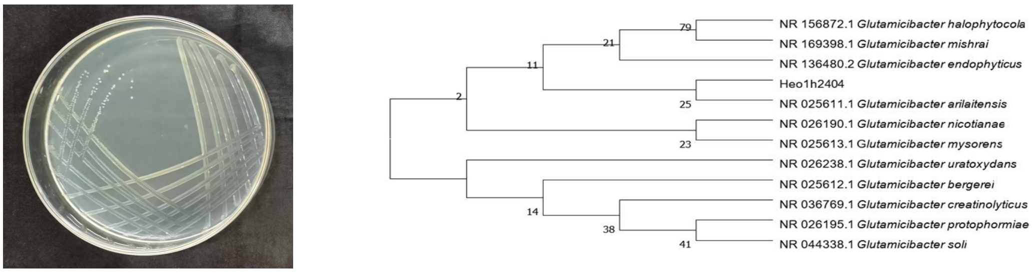

Glutamicibacter sp. Heo1h2404

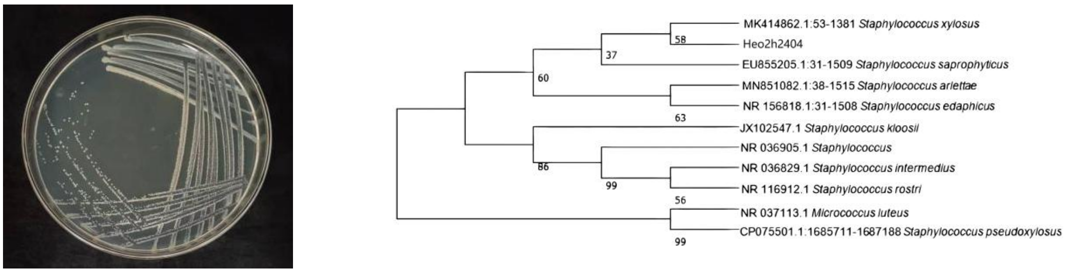

Staphylococcus xylosus Heo1h2401

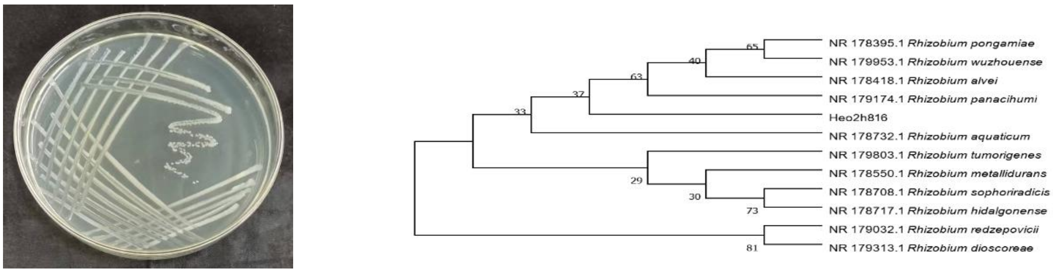

Rhizobium sp. Heo2h816

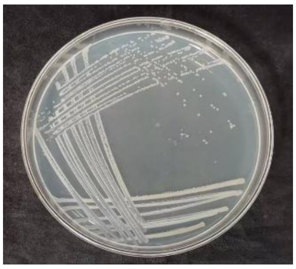

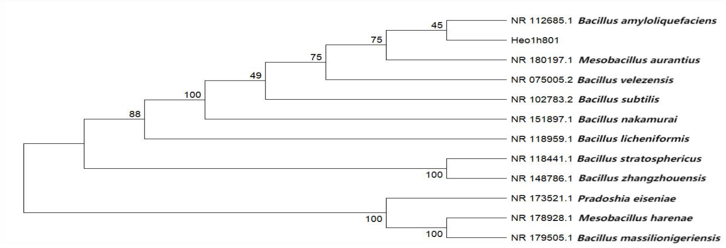

Bacillus amyloliquefaciens Heo1h801

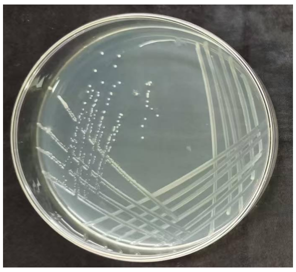

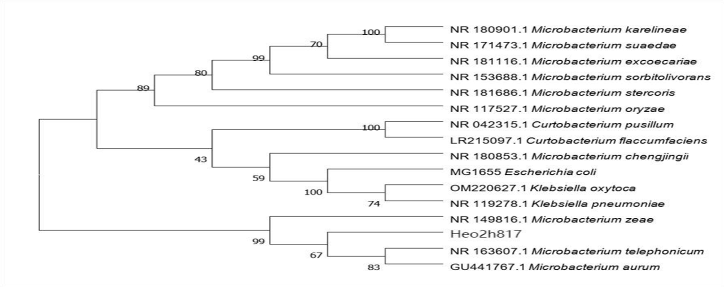

Microbacterium aurum Heo2h817
