# Supplementary material for: A Bacterium Derived from the Ovary of the Black Soldier Fly (Hermetia illucens) Attract Oviposition of the Host
Source: Biology (Basel). 2025 Aug 22;14(9):1107. doi: 10.3390/biology14091107 (PMC12467855; doi:10.3390/biology14091107)
Supplement: Supplementary file 1 [file biology-14-01107-s001.zip › Figure S2 Sequences comparison of 16S rDNA of isolate Hei101 and Hei102.pdf]

```
      v1420     v1410     v1400     v1390     v1380     v1370     v1360     v1350     v1340     v1330     v1320
GCTTACACATGCAAGTCGAGCGGTAGCACAGGGGAGCTTGCTCCCCGGGTGACGAGCGGCGGACGGGTGAGTAATGTCTGGGAAACTGCCTGATGGAGGGGGATAACTAC
||||||||||||| ||||||||||||||||||||||||||||||||||||||||||||||||||||||||||||||||||||||||||||||||||||||||||||||||
GCTTACACATGCA-GTCGAGCGGTAGCACAGGGGAGCTTGCTCCCCGGGTGACGAGCGGCGGACGGGTGAGTAATGTCTGGGAAACTGCCTGATGGAGGGGGATAACTAC
    ^10       ^20       ^30       ^40       ^50       ^60       ^70       ^80       ^90      ^100      ^110
      v1310     v1300     v1290     v1280     v1270     v1260     v1250     v1240     v1230     v1220     v1210
TGGAAACGGTAGCTAATACCGCATAACGTCGCAAGACCAAAGAGGGGGACCTTCGGGCCTCTTGCCATCAGATGTGCCCAGATGGGATTAGCTAGTAGGTGGGGTAATGG
||||||||||||||||||||||||||||||||||||||||||||||||||||||||||||||||||||||||||||||||||||||||||||||||||||||||||||||
TGGAAACGGTAGCTAATACCGCATAACGTCGCAAGACCAAAGAGGGGGACCTTCGGGCCTCTTGCCATCAGATGTGCCCAGATGGGATTAGCTAGTAGGTGGGGTAATGG
    ^120      ^130      ^140      ^150      ^160      ^170      ^180      ^190      ^200      ^210      ^220
      v1200     v1190     v1180     v1170     v1160     v1150     v1140     v1130     v1120     v1110     v1100
CTCACCTAGGCGACGATCCCTAGCTGGTCTGAGAGGATGACCAGCCACACTGGAACTGAGACACGGTCCAGACTCCTACGGGAGGCAGCAGTGGGGAATATTGCACAATG
||||||||||||||||||||||||||||||||||||||||||||||||||||||||||||||||||||||||||||||||||||||||||||||||||||||||||||||
CTCACCTAGGCGACGATCCCTAGCTGGTCTGAGAGGATGACCAGCCACACTGGAACTGAGACACGGTCCAGACTCCTACGGGAGGCAGCAGTGGGGAATATTGCACAATG
    ^230      ^240      ^250      ^260      ^270      ^280      ^290      ^300      ^310      ^320      ^330
      v1090     v1080     v1070     v1060     v1050     v1040     v1030     v1020     v1010     v1000      v990
GGCGCAAGCCTGATGCAGCCATGCCGCGTGTGTGAAGAAGGCCTTCGGGTTGTAAAGCACTTTCAGCGAGGAGGAAGGTGGTGAGCTTAATACGCTCATCAATTGACGTT
||||||||||||||||||||||||||||||||||||||||||||||||||||||||||||||||||||||||||||||||||||||||||||||||||||||||||||||
GGCGCAAGCCTGATGCAGCCATGCCGCGTGTGTGAAGAAGGCCTTCGGGTTGTAAAGCACTTTCAGCGAGGAGGAAGGTGGTGAGCTTAATACGCTCATCAATTGACGTT
    ^340      ^350      ^360      ^370      ^380      ^390      ^400      ^410      ^420      ^430      ^440
      v980      v970      v960      v950      v940      v930      v920      v910      v900      v890      v880
ACTCGCAGAAGAAGCACCGGCTAACTCCGTGCCAGCAGCCGCGGTAATACGGAGGGTGCAAGCGTTAATCGGAATTACTGGGCGTAAAGCGCACGCAGGCGGTTTGTTAA
||||||||||||||||||||||||||||||||||||||||||||||||||||||||||||||||||||||||||||||||||||||||||||||||||||||||||||||
ACTCGCAGAAGAAGCACCGGCTAACTCCGTGCCAGCAGCCGCGGTAATACGGAGGGTGCAAGCGTTAATCGGAATTACTGGGCGTAAAGCGCACGCAGGCGGTTTGTTAA
    ^450      ^460      ^470      ^480      ^490      ^500      ^510      ^520      ^530      ^540      ^550
      v870      v860      v850      v840      v830      v820      v810      v800      v790      v780      v770
GTCAGATGTGAAATCCCCGGGCTCAACCTGGGAACTGCATTTGAAACTGGCAAGCTAGAGTCTCGTAGAGGGGGGTAGAATTCCAGGTGTAGCGGTGAAATGCGTAGAGA
||||||||||||||||||||||||||||||||||||||||||||||||||||||||||||||||||||||||||||||||||||||||||||||||||||||||||||||
GTCAGATGTGAAATCCCCGGGCTCAACCTGGGAACTGCATTTGAAACTGGCAAGCTAGAGTCTCGTAGAGGGGGGTAGAATTCCAGGTGTAGCGGTGAAATGCGTAGAGA
    ^560      ^570      ^580      ^590      ^600      ^610      ^620      ^630      ^640      ^650      ^660
      v760      v750      v740      v730      v720      v710      v700      v690      v680      v670      v660
TCTGGAGGAATACCGGTGGCGAAGGCGGCCCCCTGGACGAAGACTGACGCTCAGGTGCGAAAGCGTGGGGAGCAAACAGGATTAGATACCCTGGTAGTCCACGCTGTAAA
||||||||||||||||||||||||||||||||||||||||||||||||||||||||||||||||||||||||||||||||||||||||||||||||||||||||||||||
TCTGGAGGAATACCGGTGGCGAAGGCGGCCCCCTGGACGAAGACTGACGCTCAGGTGCGAAAGCGTGGGGAGCAAACAGGATTAGATACCCTGGTAGTCCACGCTGTAAA
    ^670      ^680      ^690      ^700      ^710      ^720      ^730      ^740      ^750      ^760      ^770
      v650      v640      v630      v620      v610      v600      v590      v580      v570      v560      v550
CGATGTCGATTTGGAGGTTGTGCCCTTGAGGCGTGGCTTCCGGAGCTAACGCGTTAAATCGACCGCCTGGGGAGTACGGCCGCAAGGTTAAAACTCAAATGAATTGACGG
||||||||||||||||||||||||||||||||||||||||||||||||||||||||||||||||||||||||||||||||||||||||||||||||||||||||||||||
CGATGTCGATTTGGAGGTTGTGCCCTTGAGGCGTGGCTTCCGGAGCTAACGCGTTAAATCGACCGCCTGGGGAGTACGGCCGCAAGGTTAAAACTCAAATGAATTGACGG
    ^780      ^790      ^800      ^810      ^820      ^830      ^840      ^850      ^860      ^870      ^880
      v540      v530      v520      v510      v500      v490      v480      v470      v460      v450      v440
GGGCCCGCACAAGCGGTGGAGCATGTGGTTTAATTCGATGCAACGCGAAGAACCTTACCTACTCTTGACATCCAGAGAACTTAGCAGAGATGGTTTGGTGCCTTCGGGAA
||||||||||||||||||||||||||||||||||||||||||||||||||||||||||||||||||||||||||||||||||||||||||||||||||||||||||||||
GGGCCCGCACAAGCGGTGGAGCATGTGGTTTAATTCGATGCAACGCGAAGAACCTTACCTACTCTTGACATCCAGAGAACTTAGCAGAGATGGTTTGGTGCCTTCGGGAA
    ^890      ^900      ^910      ^920      ^930      ^940      ^950      ^960      ^970      ^980      ^990
      v430      v420      v410      v400      v390      v380      v370      v360      v350      v340      v330
CTCTGAGACAGGTGCTGCATGGCTGTCGTCAGCTCGTGTTGTGAAATGTTGGGTTAAGTCCCGCAACGAGCGCAACCCTTATCCTTTGTTGCCAGCGGTTCGGCCGGGAA
||||||||||||||||||||||||||||||||||||||||||||||||||||||||||||||||||||||||||||||||||||||||||||||||||||||||||||||
CTCTGAGACAGGTGCTGCATGGCTGTCGTCAGCTCGTGTTGTGAAATGTTGGGTTAAGTCCCGCAACGAGCGCAACCCTTATCCTTTGTTGCCAGCGGTTCGGCCGGGAA
    ^1000     ^1010     ^1020     ^1030     ^1040     ^1050     ^1060     ^1070     ^1080     ^1090     ^1100
CTCAAAGGAGACTGCCAGTGATAAACTGGAGGAAGGTGGGGATGACGTCAAGTCATCATGGCCCTTACGAGTAGGGCTACACACGTGCTACAATGGCGTATACAAAGAGA
||||||||||||||||||||||||||||||||||||||||||||||||||||||||||||||||||||||||||||||||||||||||||||||||||||||||||||||
CTCAAAGGAGACTGCCAGTGATAAACTGGAGGAAGGTGGGGATGACGTCAAGTCATCATGGCCCTTACGAGTAGGGCTACACACGTGCTACAATGGCGTATACAAAGAGA
    ^1110     ^1120     ^1130     ^1140     ^1150     ^1160     ^1170     ^1180     ^1190     ^1200     ^1210
      v210      v200      v190      v180      v170      v160      v150      v140      v130      v120      v110
AGCGACCTCGCGAGAGCAAGCGGACCTCATAAAGTACGTCGTAGTCCGGATTGGAGTCTGCAACTCGACTCCATGAAGTCGGAATCGCTAGTAATCGTAGATCAGAATGC
||||||||||||||||||||||||||||||||||||||||||||||||||||||||||||||||||||||||||||||||||||||||||||||||||||||||||||||
AGCGACCTCGCGAGAGCAAGCGGACCTCATAAAGTACGTCGTAGTCCGGATTGGAGTCTGCAACTCGACTCCATGAAGTCGGAATCGCTAGTAATCGTAGATCAGAATGC
    ^1220     ^1230     ^1240     ^1250     ^1260     ^1270     ^1280     ^1290     ^1300     ^1310     ^1320
      v100      v90       v80       v70       v60       v50       v40       v30       v20       v10
TACGGTGAATACGTTCCCGGGCCTTGTACACACCGCCCGTCACACCATGGGAGTGGGTTGCAAAAGAAGTAGGTAGCTTAACCTTCGGGAGGGCGCTACCACTTG
|||||||||||||||||||||||||||||||||||||||||||||||||||||||||||||||||||||||||||||||||||||||||||||||||||||||||
TACGGTGAATACGTTCCCGGGCCTTGTACACACCGCCCGTCACACCATGGGAGTGGGTTGCAAAAGAAGTAGGTAGCTTAACCTTCGGGAGGGCGCTACCACTTG
    ^1330     ^1340     ^1350     ^1360     ^1370     ^1380     ^1390     ^1400     ^1410     ^1420     ^1430
```
